# Modified Gan-Mai-Da-Zao decoction for behavioral and psychological symptoms of dementia: preclinical efficacy and molecular mechanisms

**DOI:** 10.1186/s13020-026-01415-y

**Published:** 2026-08-20

**Authors:** Qing-Qing Xu, Le-Ran Xue, Zhen Hu, Shiyan Qian, Yan-Fang Xian, Zhi-Xiu Lin

**Affiliations:** 1 School of Chinese Medicine, Faculty of Medicine, The Chinese University of Hong Kong, Hong Kong SAR, People’s Republic of China; 2 Department of Neurology, Zhejiang Provincial Hospital of Chinese Medicine, The First Affiliated Hospital of Zhejiang Chinese Medical University, Hangzhou, 310060 China; 3 The Chinese University of Hong Kong Chinese Medicine Specialty Clinic Cum Clinical Teaching and Research Centre, Faculty of Medicine, The Chinese University of Hong Kong, Hong Kong SAR, China; 4 Hong Kong Institute of Integrative Medicine, The Chinese University of Hong Kong, Hong Kong, Hong Kong SAR, People’s Republic of China

**Keywords:** Behavioral and psychological symptoms of dementia, Modified Gan-Mai-Da-Zao decoction, Behavioral pharmacology, Sleep, Depression, Anxiety

## Abstract

**Background:**

The behavioral and psychological symptoms of dementia (BPSD) affect over 90% of dementia patients with no effective therapeutic interventions. The Modified Gan-Mai-Da-Zao Decoction (M-GMDZD) is an innovative formula derived from GMDZD, a classical herbal formula commonly prescribed by Chinese medicine (CM) practitioners for psychiatric conditions.

**Aim:**

This study aimed to investigate the anti-BPSD effects such as sedative-hypnotic, anxiolytic, and antidepressant-like actions of M-GMDZD in animal models of BPSD.

**Methods:**

M-GMDZD was subjected to the high-performance liquid chromatography (HPLC) for chemical characterization. To investigate the sedative-hypnotic effect of M-GMDZD, ICR mice were intragastrically administered with M-GMDZD for 14 days, followed by intraperitoneal pentobarbital injections. The pentobarbital-induced sleep test was performed to assess the sleep-improving properties of M-GMDZD in mice, while the monoamine neurotransmitter levels in the pentobarbital-treated mouse brains were measured by ELISA. To evaluate anxiolytic and anti-depressant-like effects of M-GMDZD, the bilateral common carotid arteries occlusion combined with chronic restraint stress (BCCAO/CRS) model in rats was established. After drug treatment for 21 days, rats were subjected to behavioral tests, including open field test (OFT), novel recognition test (NORT), sucrose preference test (SPT), forced swim test (FST), and social interaction test (SIT). The brain tissues from BCCAO/CRS rats were collected for biochemical analysis.

**Results:**

M-GMDZD significantly prolonged the pentobarbital-induced sleep duration, increased the cerebral 5-hydroxytryptamine (5-HT) level and decreased the cerebral dopamine (DA) level in the pentobarbital-treated mice, showing promising sedative-hypnotic and sleep-improving effects. Moreover, M-GMDZD treatment markedly alleviated the anxiety-related behavioral impairments, ameliorated depression-like deficits, attenuated recognition memory impairments and improved social interaction in the BCCAO/CRS rats, as assessed by a battery of behavioral tests. The anxiolytic and antidepressant-like effects of M-GMDZD were partly attributed to the neurotrophin-3 (NT-3)-mediated neurotrophic mechanisms. We also found that M-GMDZD alleviated BPSD symptoms in BCCAO/CRS rats by restoring synaptic plasticity via the activation of TrkB/ERK/CREB pathway.

**Conclusions:**

This study has demonstrated for the first time that M-GMDZD exhibits potent anti-BPSD properties, including sedative, anxiolytic, and antidepressant-like effects in preclinical models, highlighting its potential as a promising, multi-target therapeutic intervention for the BPSD management.

**Supplementary Information:**

The online version contains supplementary material available at 10.1186/s13020-026-01415-y.

## Introduction

Dementia affects approximately 55 million people worldwide, and the number is projected to triple by 2050 [1]. Apart from cognitive and memory impairments, more than 90% of patients with dementia exhibit the behavioral and psychological symptoms of dementia (BPSD), encompassing a heterogeneous constellation of neuropsychiatric symptoms, including depression, anxiety, agitation, aggression, apathy, sleep disturbances, disinhibition and reduced sociability [2, 3]. BPSD can present at almost any stage of Alzheimer’s disease (AD), the most prevalent form of dementia; and in some patients, these symptoms emerge even prior to the onset of memory deficits, with the symptom severity escalating markedly as disease progresses [1, 4]. The clinical significance of BPSD extends far beyond their high prevalence. These symptoms exert profound adverse effects on both patients and their caregivers, accounting for up to 40% of the total cost of dementia care and serving as the leading precipitant for institutionalization [5]. From a healthcare utilization perspective, BPSD contribute to increased emergency department visits, hospitalizations, and premature nursing home placement, creating substantial economic burden estimated over $321 billion annually in the United States alone [6].

Currently, no FDA-approved therapeutic agent is available specifically for BPSD management. Treatment approaches rely on off-label use of anti-dementia medications (e.g., donepezil and memantine), antidepressants, and antipsychotics [7]. However, limited efficacy and significant adverse effects are often associated with these drugs, which substantially restrict their clinical use [3, 7]. Consequently, there exists a critical unmet medical need for safer and more effective interventions specifically targeting BPSD in dementia patients.

Chinese medicine (CM), a medical system with continuous practice for thousands of years in China, has developed comprehensive medical theories and accumulated substantial clinical experience in the prevention and treatment of dementia and its associated neuropsychiatric symptoms [8]. The Gan-Mai-Da-Zao Decoction (GMDZD) is a famous herbal formula extensively prescribed for psychiatric disorders in both historical and contemporary practice. Originally documented in the Synopsis of the Golden Chamber (Jin-Gui-Yao-Lue in Chinese) by the renowned physician Zhang Zhongjing during the Eastern Han dynasty (25–220 AD), GMDZD is composed of three herbal components including Glycyrrhizae Radix et Rhizoma (GRR, Gan-cao in Chinese), Hordei Fructus (HF, Xiao-mai in Chinese), and Jujubae Fructus (JF, Da-zao in Chinese). Previous studies showed that GRR could exert neuroprotective effects and ameliorate cerebral ischemic infarction in ischemic stroke models [9, 10]. JF, commonly well known as a dietary supplement, has been found to possess sedative-hypnotic and neuroprotective functions [11–13]. According to the CM theory, GMDZD possesses therapeutic properties of nourishing the heart to tranquilize the spirit, and replenishing the spleen to supplement Qi, making it a commonly prescribed treatment for various psychiatric conditions including depression, anxiety, and insomnia [14, 15]. A systematic review demonstrated a comparable antidepressant efficacy between GMDZD and conventional antidepressants in randomized clinical trials (RCTs), with GMDZD exhibiting a more favorable adverse effect profile [14]. Recent preclinical studies have further demonstrated that GMDZD exhibits significant antidepressant activity [16, 17]. Moreover, GMDZD was found to exert therapeutic effects on perimenopausal insomnia. In a clinical study, GMDZD showed a 80.0% clinical efficacy on patients with perimenopausal insomnia, which is a significantly superior outcome compared to eszopiclone, an FDA-approved hypnotic agent for insomnia treatment [18]. However, the effect of GMDZD on BPSD has yet to be investigated.

In CM practice, Albiziae Flos (AF, He-huan-hua in Chinese) is widely used for the treatment of depression, insomnia and anxiety [19, 20], while Margaritifera Concha (MC, Zhen-zhu-mu in Chinese) is commonly prescribed for insomnia [21]. Flavonol glycosides from AF, including quercitrin and isoquercitrin, showed a dose-dependent increase in the pentobarbital-induced sleep duration in mice [22]. The water-soluble, acid-soluble, and conchiolin proteins of MC were found to reduce locomotor activity and increase convulsion latency in mice through downregulation of 5-HT3 expression and upregulation of GABA_B_ levels, thereby exerting anticonvulsant and sedative-hypnotic effects [23]. To develop a more effective formulation for BPSD treatment, we proposed incorporating AF and MC into GMDZD to form a modified GMDZD (M-GMDZD). Based on the above pharmacological evidence, we hypothesize that M-GMDZD may be an effective formula with more potent anti-BPSD efficacy than that of the original GMDZD. To advance M-GMDZD as an innovative therapeutic intervention for BPSD, this study aimed to test this hypothesis using preclinical experimental BPSD models and to unravel the underlying molecular mechanisms.

## Methods

### Chemicals and reagents

Diazepam was purchased from the Vickmans Laboratories Co. Ltd (Hong Kong, China). Fluoxetine hydrochloride was purchased from the Sigma-Aldrich (St. Louis, MO, USA). Liquiritin, ammonium glycyrrhetate, and quercitrin (purity ≥ 98% for all) were purchased from Chengdu Mansite Pharmacetical Co. Ltd (Sichuan, China). All other chemicals and reagents used were of analytical grade.

### Extraction and quality control of herbal materials

The crude decoction pieces of GRR, HF, JF, AF and MC were purchased from Zhixin Pharmaceutical Co., Ltd (Guangdong, China), a Chinese herbal supplier with GMP accreditation. These herbs were authenticated by Ms. Yu-ying Zong, a senior pharmacognosist at the School of Chinese Medicine, The Chinese University of Hong Kong (CUHK), with voucher specimens deposited according to the Chinese Pharmacopoeia 2020. For the preparation of M-GMDZD extract, the five herbs (i.e. GRR, HF, JF, AF, and MC) were ground to powder or pieces and mixed in the ratio of 3:5:5:5:10. GMDZD was prepared using GRR, HF, and JF in a ratio of 3:5:5. Both formulations were prepared based on the PI’s clinical experience and in accordance with individual herbal dosages specified in the Chinese Pharmacopeia (2020). Each herbal mixture was extracted with boiling tenfold water for 1 h, followed by filtration. The aqueous extraction was repeated three times. The combined filtrates were concentrated in a rotary evaporator under negative pressure and subsequently lyophilized to produce a freeze-dried powder. The extraction yields of M-GMDZD and GMDZD were 21.2% and 33.1%, respectively. The freeze-dried aqueous extracts were stored at −20 ℃ for subsequent study.

The high-performance liquid chromatography (HPLC) profiles of M-GMDZD and GMDZD were performed using an ACQUITY UPLC system (Waters, USA) equipped with a PDA eλ detector, FTN sample manager, and quaternary solvent manager. Briefly, M-GMDZD or GMDZD was solubilized in 70% aqueous ethanol and automatically injected into the UPLC system. Separation was achieved using a Waters Spherisorb^®^ chromatographic column (4.0 mm × 150 mm, 5 μm) with a mobile phase consisting of 0.01% phosphoric acid in water (solvent A) and acetonitrile (solvent B). The elution gradient increased linearly from 10 to 60% solvent B over 30 min at a flow rate of 1.0 mL/min. Chromatographic separation was conducted at room temperature (about 25 ℃) with detection wavelengths set at 256 nm. The sample injection volume was 10 μL.

### Animals

Eight-week-old male and female ICR mice and Sprague–Dawley (SD) rats were obtained from the Laboratory Animal Services Centre, CUHK. Rodents were maintained under standardized conditions (5 animals per cage, 12 h light/dark cycle, a constant room temperature of 22 ± 2 ℃, and relative humidity 50 ± 10%) with ad libitum access to food and water. The study protocol was approved by the Animal Experimentation Ethics Committee of CUHK (Ref. No. 22-108-HMF). All efforts were made to minimize animal suffering and reduce the number of animals used.

### Establishment of BCCAO/CRS rat model

The BCCAO/CRS rat model was established as described previously with modifications [24]. Eight-week-old SD rats underwent surgical procedures under anesthesia. After a midline anterior neck incision, the right common carotid artery (CCA) was exposed and permanently occluded using 5-0 silk sutures. After operation, rats were transferred to an electric heating pad for maintenance of body temperature until recovery from anesthesia. Two days later, the left CCA was ligated using the identical procedure. For the control group, the same surgical procedures were performed but without ligation. All animals were allowed for a 14-day recovery period after the first surgery.

Beginning on day 16, rats in the BCCAO/CRS groups were subjected to restraint stress by placement in transparent plastic tubes (5.5 cm inner diameter, 25 cm length) for 3 h daily for 14 consecutive days. The restraint apparatus allowed unrestricted breathing through evenly distributed ventilation holes but limiting movement. After each restraint session, rats returned to their home cages with free access to food and water. Rats in the control group remained in cages without restraint stress exposure.

### Animal grouping and drug administration

Experiment 1: To investigate the sedative-hypnotic effects of M-GMDZD in the pentobarbital-induced sleep in ICR mice, 8-week-old male and female ICR mice (1:1) were randomly assigned into six groups (n = 10): control, GMDZD (1.9 g/kg), M-GMDZD (1.3, 2.6, and 5.2 g/kg), and diazepam (positive control, 1 mg/kg, i.p.). The doses of M-GMDZD and GMDZD were determined based on clinical usage. In clinical Chinese herbal medicine practice, M-GMDZD and GMDZD are usually prescribed at the daily doses of 84 g and 39 g raw herbal materials, respectively. The human-to-mouse dose conversion was calculated using extraction yields of 21.2% for M-GMDZD and 33.1% for GMDZD, a reference human body weight of 60 kg, and a human-to-mouse conversion factor of 9. A middle dose of 2.6 g extract/kg for M-GMDZD and 1.9 g extract/kg for GMDZD were then established. Consequently, 1.3 g/kg and 5.2 g/kg were used as the low dose and the high dose for M-GMDZD in this study, respectively. GMDZD, M-GMDZD and diazepam were dissolved in 0.9% saline. GMDZD, M-GMDZD and vehicle were administered intragastrically once daily for 14 days.

Experiment 2: To investigate the anxiolytic and anti-depressant-like effects of M-GMDZD in the BCCAO/CRS rat model, rats were randomly assigned into seven groups (n = 10): control, BCCAO/CRS plus vehicle group, BCCAO/CRS plus GMDZD (1.3 g/kg), BCCAO/CRS plus M-GMDZD (0.9, 1.8 and 3.6 g/kg), and BCCAO/CRS plus fluoxetine (positive control, 20 mg/kg). The human-to-rat dose conversion was calculated using extraction yields of 21.2% for M-GMDZD and 33.1% for GMDZD, a reference human body weight of 60 kg, and a human-to-rat conversion factor of 6. A middle dose of 1.8 g extract/kg for M-GMDZD and 1.3 g extract/kg for GMDZD were then established. Consequently, 0.9 g/kg and 3.6 g/kg were used as the low dose and the high dose for M-GMDZD, respectively. GMDZD, M-GMDZD and fluoxetine were dissolved in 0.9% saline and administered intragastrically once daily for 21 days (from day 15 to 36).

### The pentobarbital-induced sleep test

The pentobarbital-induced sleep test was performed to evaluate the sedative-hypnotic effects of M-GMDZD in mice. Briefly, 30 min after the final drug treatment, 55 mg/kg pentobarbital sodium was injected intraperitoneally to mice to induce sleep. Sleep onset was defined as the loss of righting reflex, characterized by the maintenance of a supine posture for more than 1 min. Sleep latency was measured as the time interval between the pentobarbital administration and the loss of righting reflex. The sleep duration was quantified as the period from sleep onset until the reappearance of righting reflex. The recovery time was defined as the interval from the reappearance of righting reflex until restoration of spontaneous locomotor activity. Mice that failed to lose their righting reflex within 15 min of the pentobarbital administration were excluded from the analysis.

### The open filed test (OFT)

The OFT was performed to evaluate locomotor activity and anxiety levels in BCCAO/CRS rats as described in our previous study [25] with minor modifications. Rats were individually placed in the center of an open field apparatus (100 cm × 100 cm × 50 cm) under dim lighting conditions and allowed to explore freely for 10 min. Movement routes, velocity, and time spent in the center zone were recorded and analyzed using a video tracking system (SuperMaze V2.0, China). The apparatus was thoroughly cleaned with 70% ethanol before initial use and between subsequent tests to eliminate olfactory cues.

### Novel object recognition test (NORT)

The NORT was performed to evaluate recognition memory in the BCCAO/CRS rat model as described in our previous study [25] with minor modification. The test was conducted in an open-field arena (100 cm × 100 cm × 50 cm, identical to the OFT apparatus) over three consecutive days. On day 1, rats were habituated to the arena by allowing 10 min of free exploration (no objects in the arena, these data were utilized for open field analyses). On day 2, rats were placed in the arena containing two identical objects (purple plastic cylinders, 8 cm in diameter, 17 cm in height, designated as object A) and allowed to explore for 10 min. On day 3, the object A was replaced with a novel object of different color and shape (a glass container filled with crystal violet solution, 7 × 7 × 18 cm, designated as object B), and rats were then allowed 10 min of free exploration. The apparatus and objects were thoroughly cleaned with 70% ethanol before initial use and between subsequent tests to eliminate olfactory cues. Exploratory behavior was defined as the rat directing its nose toward an object at a distance ≤ 2 cm, and was recorded using a video tracking system (SuperMaze V2.0 software, China). The recognition index was calculated using the equation [(Time exploring novel object)/(Time exploring novel object + Time exploring familiar object)].

### The sucrose preference test (SPT)

The SPT was performed to assess the anhedonia-like behavior. Briefly, rats were subjected to a 48-h habituation period, during which two bottles containing 1% sucrose solution (w/v) were placed in each cage for the first 24 h. For the subsequent 24 h, one bottle was replaced with water to familiarize the animals with both solutions. After habituation, rats were deprived of food and water for 24. For the test phase, rats were individually housed in cages equipped with two pre-weighed bottles containing 100 mL of water and 100 mL of 1% sucrose solution (w/v), respectively. After a 6-h test period, the remaining volume in each bottle was measured to calculate fluid consumption. The sucrose preference was calculated as the percentage of sucrose solution consumed relative to the total fluid intake [(sucrose consumption/total fluid consumption) × 100%].

### The social interaction test (SIT)

The SIT was conducted to assess sociability and social preference in the BCCAO/CRS rat model. The apparatus consisted of a rectangular Plexiglas box (120 cm × 40 cm × 40 cm) divided into three equal chambers (i.e., left, center, and right chambers, each 40 cm × 40 cm × 40 cm) by transparent walls with openings doors (10 cm × 10 cm). Rats were habituated to the examining room for 1 h before the test. The SIT consisted of three consecutive phases, including apparatus habituation, sociability assessment, and social novelty preference assessment. During the habituation phase, rats was placed in the center chamber and allowed to freely explore all three empty chambers for 10 min. For the sociability phase, a novel conspecific rat (Stranger 1) that had no prior contact with the test rat was placed inside a cylindrical wire mesh cage (13 cm in diameter, 21 cm in height) in one of the side chambers (left chamber), while an identical empty mesh cage was placed in the opposite chamber (right chamber). The mesh cages allowed visual, olfactory, and limited tactile contact while preventing aggressive interactions. The test rat was then placed in the center chamber and allowed to freely explore all three chambers for 5 min. Immediately following the sociability phase, the social novelty preference phase was initiated by placing a second novel conspecific rat (Stranger 2) in the previously empty mesh cage. The test rat was allowed to explore all three chambers for an additional 5 min, with free access to both Stranger 1 (now familiar) and Stranger 2 (novel). A video tracking system was used to record the time spent in each chamber, the time spent in close proximity (defined as ≤ 5 cm) to each wire-cage, the number of entries into each chamber, and the first sniffing time to stranger rats in BCCAO/CRS rats. Sociability was defined as significantly more time spent with Stranger 1 compared to the empty cage. Social novelty preference was defined as significantly more time spent with Stranger 2 compared to Stranger 1.

### The forced swim test (FST)

The FST was conducted to assess depressive-like behavior in the BCCAO/CRS rats. Briefly, each rat was gently placed in a transparent cylindrical glass vessel (20 cm in diameter, 50 cm in height) filled with water (35 cm depth, 24 ± 1 ℃) and allowed to swim for 6 min. The total duration of immobility, defined as the absence of active escape-directed behaviors (such as swimming or climbing), was measured. Immobility was considered when the rat remained floating in the water, making only the movements necessary to keep its head above the surface. All testing sessions were video-recorded. Only data from the final 4 min of the 6-min test period were analyzed, as the first 2 min are considered a habituation period.

### Preparation of brain tissue samples

Mice or rats under deep anesthesia were sacrificed 24 h after the behavioral tests. Animals were transcardially perfused with pre-chilled normal saline until clear fluid emerged from the right atrium. The brain tissues were then isolated on ice and stored at −80 ℃ until further use.

### Measurement of monoamine neurotransmitter levels

The cerebral levels of norepinephrine (NE), 5-hydroxytryptamine (5-HT), dopamine (DA) and γ-aminobutyric acid (GABA) in the pentobarbital-treated mice were assessed using commercially available ELISA kit (Mlbio, Shanghai, China) according to the manufacturer’s protocol.

### RNA-seq and bioinformatics analysis

Total RNA was extracted from the prefrontal cortex of rats in BCCAO/CRS and BCCAO/CRS + M-GMDZD-H groups (n = 3) using Trizol reagent (Invitrogen, USA). RNA sequencing libraries were constructed using VAHTS Universal V10 RNA-seq Library Prep Kit (Premixed Version) and sequenced on an Illumina Novaseq 6000 platform by Oebiotech Co., Ltd (Shanghai, China). Differential expression analysis was conducted using DESeq2.

### Measurement of neurotrophic factors levels

The cerebral levels of the brain-derived neurotrophic factor (BDNF), neurotrophin-3 (NT-3), and nerve growth factor (NGF) in the BCCAO/CRS rats were evaluated using commercially available ELISA kit (Mlbio, Shanghai, China) according to the manufacturer’s protocol.

### Western blot analysis

The brain tissues were homogenized in the pre-chilled RIPA lysis buffer (Cat. 89901, Thermo Fisher Scientific, USA) containing 1 × protease/phosphatase inhibitor cocktail (Cat. 78442, Thermo Fisher Scientific, USA) to prepare 10% (w/v) homogenates. After centrifugation at 13,000 rpm at 4 °C for 20 min, the supernatants were collected. The protein concentrations were quantified using the BCA assay method. Equal amounts (30 μg) of denatured protein samples were separated on the SDS–polyacrylamide gels and subsequently transferred to the polyvinyl difluoride (PVDF) membranes. After blocking with 5% non-fat milk, the membranes were incubated overnight at 4 ℃ with primary antibodies, including GluN2A (1:1000, 4205S, Cell Signaling Technology), GluN2B (1:1000, 4212S, Cell Signaling Technology), GluA1 (1:1000, 13185S, Cell Signaling Technology), GluA2 (1:1000, 13607S, Cell Signaling Technology), PSD93 (1:1000, 19046S, Cell Signaling Technology), PSD95 (1:1000, 3450S, Cell Signaling Technology), SYN1 (1:1000, 5297S, Cell Signaling Technology), synaptotagmin (SYT) (1:1000, 3347S, Cell Signaling Technology), p-TrkB (Y-705) (1:1000, ab229908, Abcam), TrkB (1:1000, ab18987, Abcam), p-ERK (42/44) (1:1000, 4370S, Cell Signaling Technology), ERK (42/44) (1:1000, 9102S, Cell Signaling Technology), p-CREB (1:5000, 28792-1-AP, Protintech), CREB (1:5000, 12208-1-AP, Protintech), and GAPDH (1:10000, ab181602, Abcam). Following primary antibody incubation, the membranes were probed with anti-mouse or anti-rabbit IgG secondary antibody (Thermo Fisher Scientific, USA) for 2 h at room temperature. The protein bands were visualized using ECL reagent (Cat. 34580, Thermo Scientific). The densitometric analysis was performed using ImageJ software (NIH, Bethesda, MD, USA).

### Statistical analysis

Data were presented as means ± standard error of the mean (SEM). Differences among multiple groups were analyzed using one-way ANOVA followed by Tukey’s post-hoc test for multiple comparisons. Comparisons between two groups were performed using t-test. Statistically significant was defined as *p* < 0.05.

## Results

### The HPLC profiles of GMDZD and M-GMDZD

**Fig. 1:**
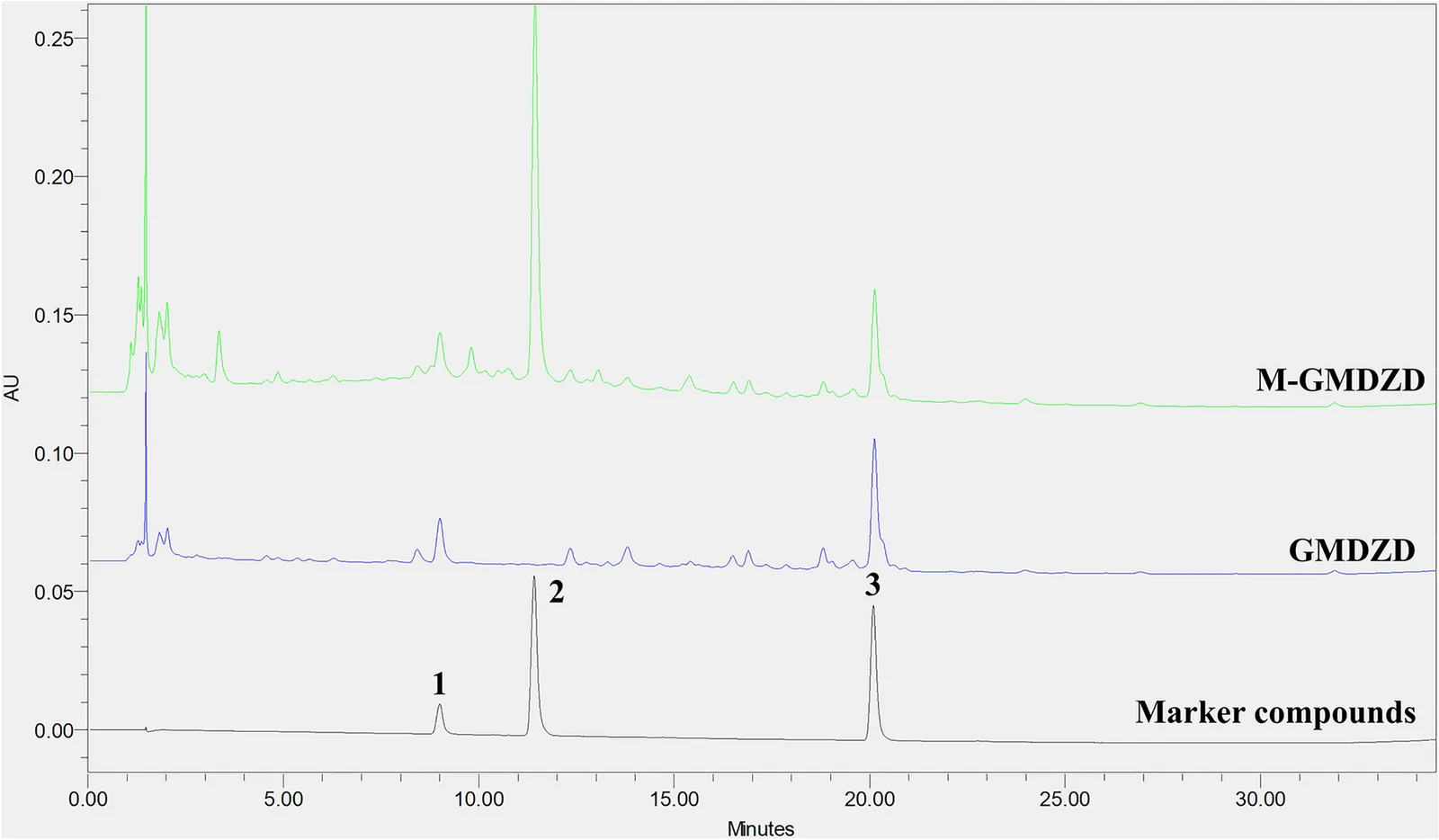
The HPLC profiles of the aqueous ethanol extracts of M-GMDZD and GMDZD. 1: liquiritin; 2: quercitrin; 3: ammonium glycyrrhetate

Three representative compounds, including liquiritin, ammonium glycyrrhetate, and quercitrin, were served as chemical markers for the quality control of M-GMDZD and GMDZD. All compounds demonstrated robust linear relationships (r^2^ ≥ 0.9995). According to the Chinese Pharmacopoeia 2020, the content of glycyrrhizic acid = the content of ammonium glycyrrhizate/1.0207. Based on the calibration curves derived from the standard substances, the contents of the marker compounds in M-GMDZD and GMDZD were determined as follows: M-GMDZD contains 0.338% liquiritin, 0.685% ammonium glycyrrhetate (equivalent to 0.671% glycyrrhizic acid), and 0.627% quercitrin. GMDZD, which lacks AF (the botanical source of quercitrin), contains 0.566% liquiritin and 1.266% ammonium glycyrrhetate (equivalent to 1.241% glycyrrhizic acid). The HPLC chromatograms of both formulations were shown in Fig. 1.

### Effects of M-GMDZD on the pentobarbital-induced sleeping time in mice

**Fig. 2:**
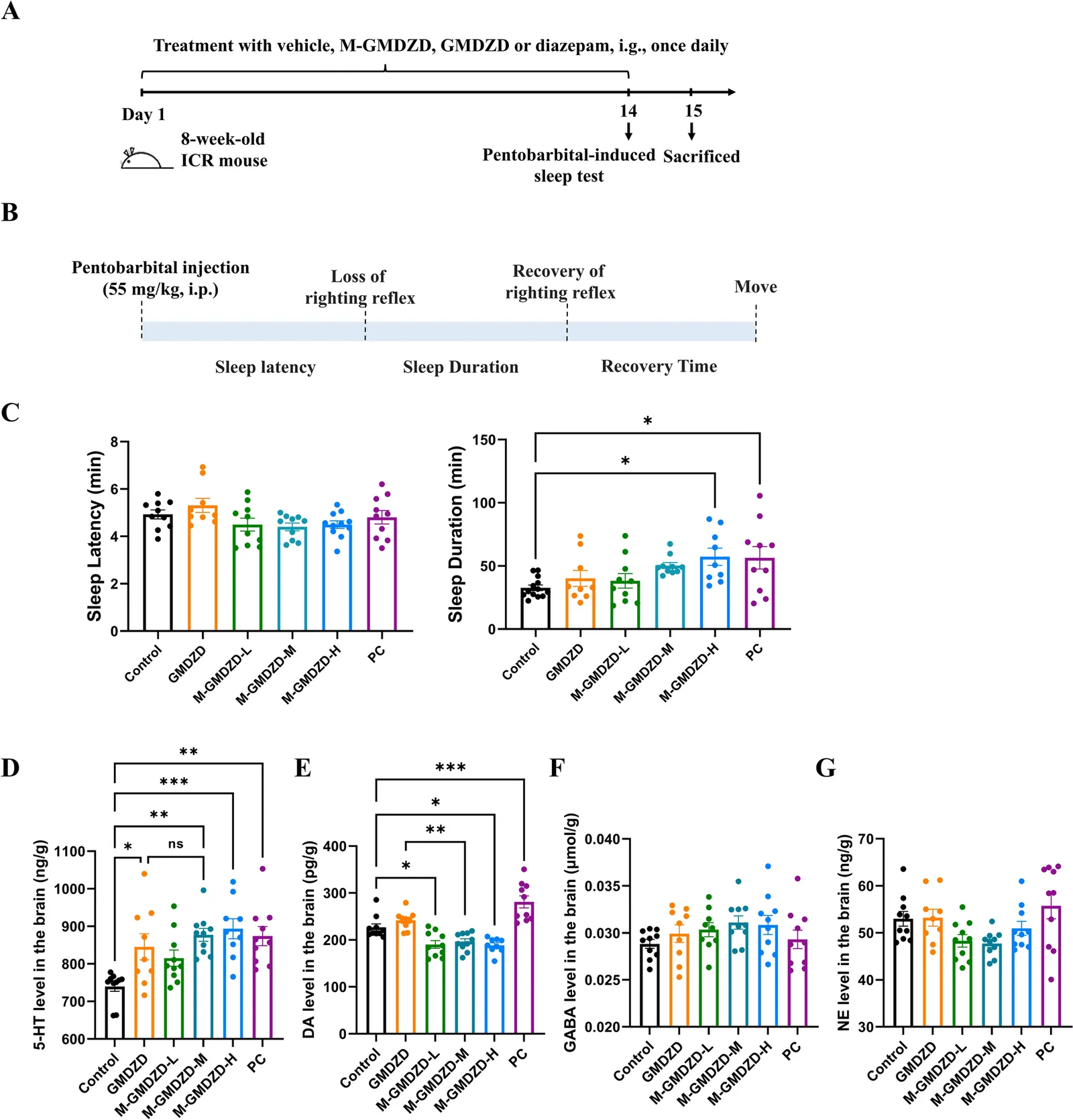
Evaluation of the sedative-hypnotic effects of M-GMDZD in the pentobarbital-induced sleeping time in mice (n = 10). **A** Experimental design and treatment schedule for evaluating the sedative-hypnotic effects of M-GMDZD in ICR mice. **B** Experimental design of the pentobarbital-induced sleep test. **C** The pentobarbital-induced sleep latency and sleep duration. **D**–**G** Effects of M-GMDZD on monoamine neurotransmitter level in the brain tissues of the pentobarbital-treated mice. **D** Cerebral 5-HT level; **E** cerebral DA level; **F** cerebral NE level; **G** cerebral GABA level. Data were expressed as the mean ± SEM. *p < 0.05, **p < 0.01 and ***p < 0.001 compared with the control group. *PC* positive control, i.e., diazepam

To evaluate the sedative-hypnotic effect of M-GMDZD treatment, the pentobarbital-induced sleep test was applied in this experiment. There were no significant differences among all groups in the sleep latency (Fig. 2C). Compared with the control group, the administration of M-GMDZD at the high dose (5.2 g/kg) and diazepam could markedly prolong the pentobarbital-induced sleep duration in mice (*p* < 0.05 for both, Fig. 2C). Treatments with M-GMDZD at the low and medium doses (1.3 and 2.6 g/kg, respectively) and GMDZD showed a trend of increase in the sleep duration in mice, as compared with the control group. However, the differences were not statistically significant. These results demonstrated that M-GMDZD possesses the sedative-hypnotic and sleep-improving effects in mice.

### Effects of M-GMDZD on the monoamine neurotransmitter levels in the brain tissues of the pentobarbital-treated mice

To further investigate the mechanisms underlying the sedative-hypnotic effects of M-GMDZD, we evaluated monoamine neurotransmitter levels in the brains of the pentobarbital-treated mice. As shown in Fig. 2D, M-GMDZD at the medium and high doses could significantly accentuate the 5-HT levels in the brain tissues of the pentobarbital-treated mice (*p* < 0.01 and *p* < 0.001, respectively), as compared with the control group. Moreover, mice in the GMDZD and diazepam groups exhibited markedly higher cerebral levels of 5-HT than those in the control group (*p* < 0.05 and *p* < 0.01, respectively). As shown in Fig. 2E, when compared with the control group, the administration of M-GMDZD could markedly attenuate the DA level in the brain tissues of the pentobarbital-treated mice (*p* < 0.05 for M-GMDZD at low dose; *p* < 0.01 for M-GMDZD at medium dose; *p* < 0.05 for M-GMDZD at high dose). Treatment with diazepam also efficiently reduced the cerebral DA level in the pentobarbital-treated mice (*p* < 0.001), as compared with the control group. In addition, the administration of M-GMDZD at all doses showed a trend of reduction in the cerebral NE level in mice when compared with the control group (Fig. 2F), but no statistic differences in the cerebral NE level were found among all groups. There were no significant differences in the cerebral GABA level among all groups (Fig. 2G).

### Effects of M-GMDZD on the behavioral impairments in the BCCAO/CRS rats

#### Effects of M-GMDZD on the locomotor activity and the anxiety-related behavior in BCCAO/CRS rats

**Fig. 3:**
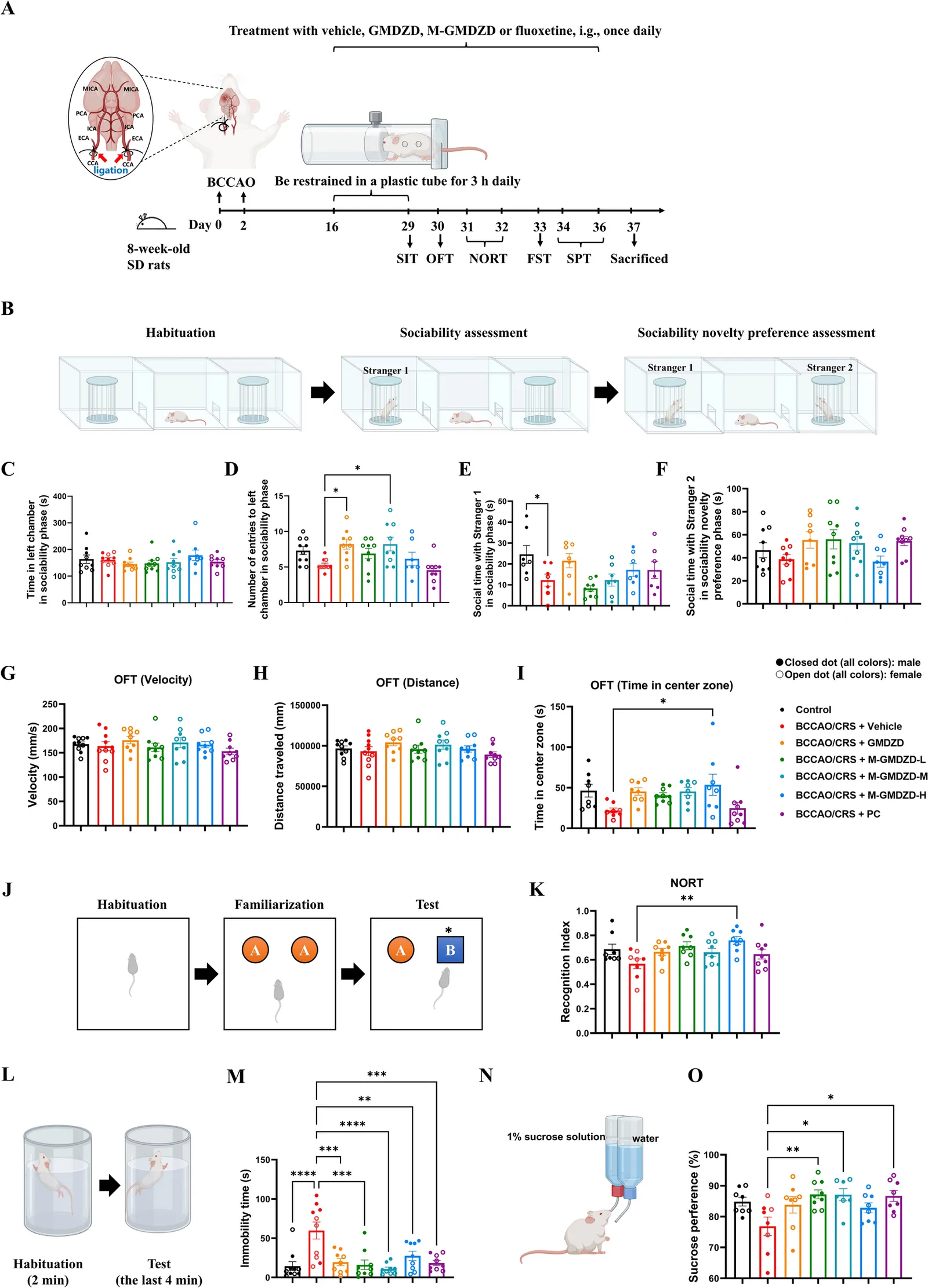
Effects of M-GMDZD on the behavioral impairments in BCCAO/CRS rats (n = 10). **A** Experimental design and treatment schedule for evaluating the anxiolytic and anti-depressant-like effects of M-GMDZD in BCCAO/CRS rats. **B** Experimental design of SIT. **C** Time in the left chamber in sociability phase in SIT. **D** Number of entries to the left chamber in sociability phase in SIT. **E** Social time with Stranger 1 in sociability phase in SIT. **F** Social time with Stranger 2 in sociability novelty preference phase. **G**–**I** Effects of M-GMDZD on the locomotor activity and the anxiety-related behavior in BCCAO/CRS rats. **G** velocity in OFT; **H** distance traveled in OFT; **I** time spent in the center zone in OFT. **J** Experimental design of NORT. **K** Recognition index in NORT. **L** Experimental design of FST. **M** Immobility time in FST. **N** Experimental design of SPT. **O** Sucrose preference in SPT. Data were expressed as the mean ± SEM. *p < 0.05, **p < 0.01, ***p < 0.001 and ****p < 0.0001, compared with the BCCAO/CRS + vehicle group. *PC* positive control, i.e., fluoxetine

The OFT was performed to evaluate anxiety-related behavior and locomotor activity in BCCAO/CRS rats. As shown in Fig. 3G–I, in the OFT, no statistically significant differences were found in the movement velocity and distance travelled among all groups, showing that BCCAO/CRS did not induce motor coordination deficit. Rats in the BCCAO/CRS + vehicle group spent less time exploring the center of the chamber than those in the control group. However, this difference was not significant. High dose M-GMDZD treatment significantly increased the time exploring the center zone in the BCCAO/CRS rats (*p* < 0.05), as compared with the BCCAO/CRS + vehicle group. In addition, BCCAO/CRS rats treated with GMDZD and M-GMDZD at low and medium doses exhibited increased exploration time in the center zone compared to the BCCAO/CRS + vehicle group, although these differences were not statistically significant.

#### Effects of M-GMDZD on the recognition memory in BCCAO/CRS rats

The NORT was performed to evaluate the recognition memory in BCCAO/CRS rats. This test leverages the innate proclivity of rats to preferentially investigate a novel object over a familiar one. As shown in Fig. 3K, the BCCAO/CRS + vehicle group exhibited a lower recognition index than the control group, indicating that BCCAO/CRS may impair the ability to discriminate between familiar and novel objects in rats. Treatment with M-GMDZD at high dose significantly increased the recognition index in the BCCAO/CRS rats (*p* < 0.05), when compared with the BCCAO/CRS + vehicle group, demonstrating that M-GMDZD could alleviate the recognitive memory deficits in BCCAO/CRS rats.

#### Effects of M-GMDZD on FST in BCCAO/CRS rats

The FST was performed to evaluate the effects of M-GMDZD on depression-like behavior in BCCAO/CRS rats. As shown in Fig. 3L, M, BCCAO/CRS markedly increased the immobility time in FST (*p* < 0.0001), as compared with the control group, indicating that BCCAO/CRS could induce depressive-like behavior in rats. All treatments, including GMDZD, M-GMDZD and fluoxetine, effectively decreased the immobility time (*p* < 0.01 for M-GMDZD at high dose; *p* < 0.001 for GMDZD, M-GMDZD at low dose and fluoxetine; *p* < 0.0001 for M-GMDZD at medium dose), when compared with the BCCAO/CRS + vehicle group.

#### Effects of M-GMDZD on SIT in BCCAO/CRS rats

The SIT was performed to evaluate the effects of M-GMDZD on sociability and social preference in BCCAO/CRS rats. As shown in Fig. 3B–F, no significant difference was found in the time in the left chamber among all groups in sociability phase. When compared with the BCCAO/CRS + vehicle group, the administration of GMDZD and M-GMDZD at medium dose significantly increased the number of entries into the left chamber in BCCAO/CRS rats (*p* < 0.05 for both) in sociability phase. In sociability phase, rats in the BCCAO/CRS group spent significantly less social time with stranger 1 (*p* < 0.05) than those in the control group, indicating that BCCAO/CRS could induce social impairments in rats. Treatments with M-GMDZD showed a dose-dependent increase in the social time with stranger 1 in BCCAO/CRS rats. Moreover, the administration of GMDZD and M-GMDZD at high dose showed a trend of increase in the social time with stranger 1, as compared with the BCCAO/CRS group. However, the differences were not statistically significant. In sociability novelty preference phase, treatments with GMDZD and M-GMDZD showed a trend of increase on the social time with stranger 2, but the difference was not statistically significant, as compared with the BCCAO/CRS group.

#### Effects of M-GMDZD on SPT in BCCAO/CRS rats

The SPT was performed to evaluate the effects of M-GMDZD on anhedonia, a key feature of depression in dementia, in BCCAO/CRS rats. As shown in Fig. 3N, O, rats in the BCCAO/CRS + vehicle group preferred less to sucrose solution than those in the control group; however, this difference was not significant. The administration of M-GMDZD at low and medium doses and fluoxetine markedly increased the sucrose preference in BCCAO/CRS rats (*p* < 0.05 for M-GMDZD at medium dose and fluoxetine; *p* < 0.01 for M-GMDZD at low dose), when compared with the BCCAO/CRS + vehicle group.

### RNA-seq analysis reveals neuronal signaling- and synaptic function-related transcriptomic alterations in the M-GMDZD group

Transcriptomic sequencing analysis was performed comparing the M-GMDZD-treated and the BCCAO/CRS groups, identifying a total of 265 differentially expressed genes (DEGs) (Fig. S1A). GO enrichment analysis showed that the DEGs were enriched in pathways related to neuronal signaling and synaptic regulation, including the neuropeptide signaling pathway, dopaminergic synapse, axon terminus, synaptic vesicle membrane, axon, and dopamine neurotransmitter receptor activity (Fig. S1B). KEGG pathway classification and KEGG enrichment analyses further demonstrated that the altered transcripts were predominantly associated with signal transduction and nervous system-related processes (Fig. S1C), with enrichment in the cAMP signaling pathway, neuroactive ligand-receptor interaction, cholinergic synapse, and dopaminergic synapse (Fig. S1D).

### Effects of M-GMDZD on neurotrophic factors in the brains of BCCAO/CRS rats

**Fig. 4:**
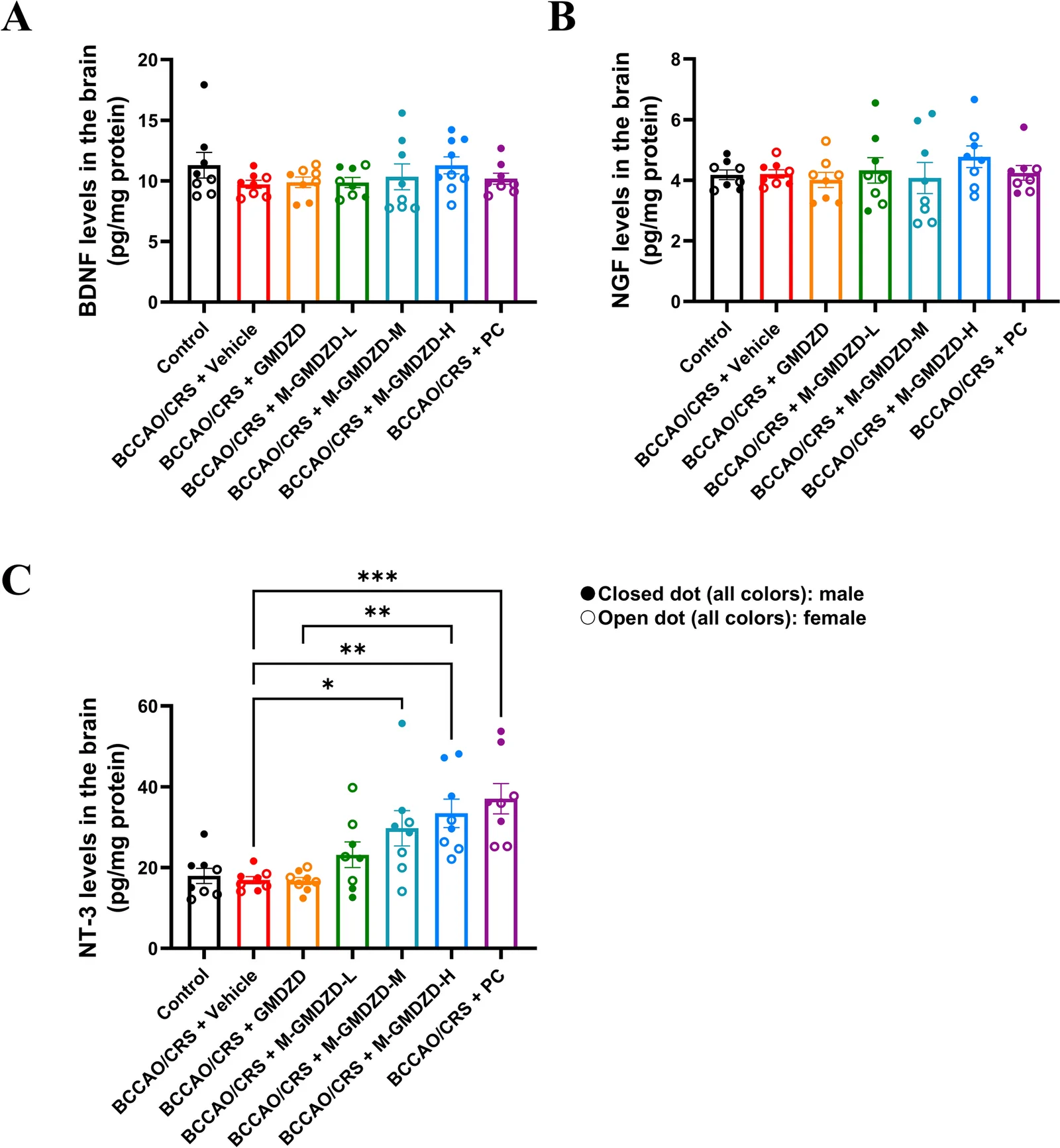
Effects of M-GMDZD on the neurotrophic factors in the brains of BCCAO/CRS rats (n = 8). **A** BDNF level in the brain. **B** NGF level in the brain. **C** NT-3 level in the brain. Data were expressed as the mean ± SEM. *p < 0.05, **p < 0.01 and ***p < 0.001, compared with the BCCAO/CRS + vehicle group

The cerebral levels of neurotrophic factors were then evaluated in BCCAO/CRS rats. As shown in Fig. 4, no significant differences were found in the cerebral levels of BDNF and NGF among all groups. When compared with the BCCAO/CRS + vehicle group, treatments with M-GMDZD at medium and high doses and fluoxetine significantly increased the NT-3 levels in the brain tissues of BCCAO/CRS rats (*p* < 0.05 for M-GMDZD at medium dose; *p* < 0.01 for M-GMDZD at high dose; p < 0.001 for fluoxetine). Furthermore, the high dose M-GMDZD group had markedly higher cerebral NT-3 levels than the GMDZD group (*p* < 0.01).

### Effects of M-GMDZD on the synaptic plasticity in the brains of BCCAO/CRS rats

**Fig. 5:**
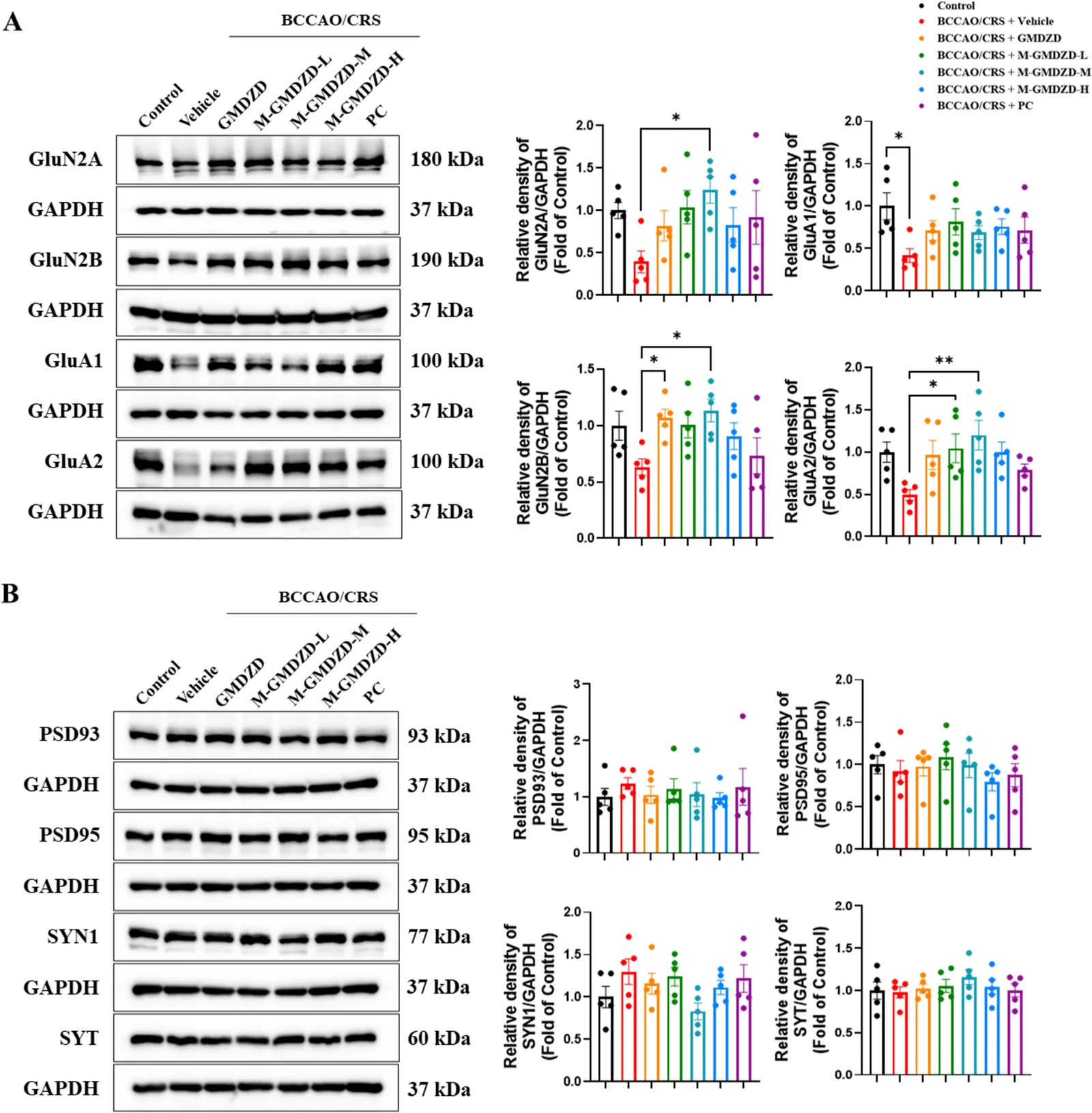
Effects of M-GMDZD on the synaptic plasticity in the brains of BCCAO/CRS rats (n = 5). **A** representative western blotting images and quantitative analysis of the protein expressions of GluN2A, GluN2B, GluA1 and GluA2. **B** representative western blotting images and quantitative analysis of the protein expressions of PSD93, PSD95, SYN1 and SYT. Data were expressed as the mean ± SEM. *p < 0.05 and **p < 0.01, compared with the BCCAO/CRS + vehicle group

As shown in Fig. 5, the western blot results demonstrated that the treatment with M-GMDZD at medium dose could significantly increase the cerebral protein expression of GluN2A in BCCAO/CRS rats (*p* < 0.05). The administration of GMDZD and M-GMDZD at medium dose markedly increase the cerebral protein level of GluN2B in BCCAO/CRS rats (*p* < 0.05 for both). Moreover, rats in the BCCAO/CRS + M-GMDZD-L group and the BCCAO/CRS + M-GMDZD-M group had significant higher cerebral protein expression of GluA2 than those in the BCCAO/CRS group (*p* < 0.05 for M-GMDZD at low dose and *p* < 0.01 for M-GMDZD at medium dose). We did not observe statistically significant changes in the protein expressions of GluA1, PSD93, PSD95, SYN1 and SYT among all groups within the limits of our detection.

### Effects of M-GMDZD on TrkB/ERK/CREB pathway in the brain tissues of BCCAO/CRS rats

**Fig. 6:**
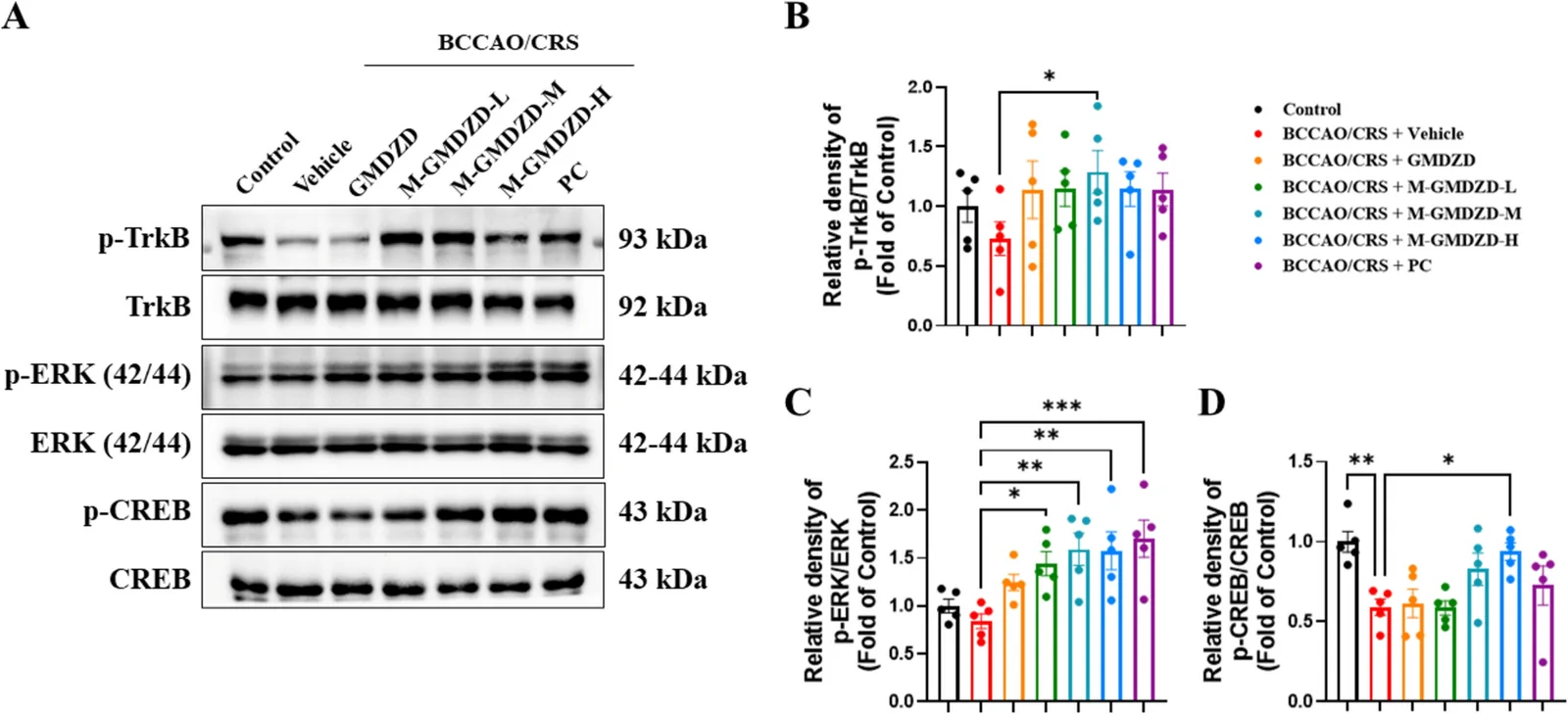
Effects of M-GMDZD on the TrkB/ERK/CREB pathway in the brains of BCCAO/CRS rats (n = 5). **A** representative western blotting images. **B** quantitative analysis of the protein expressions of p-TrkB/TrkB. **C** quantitative analysis of the protein expressions of p-ERK(42/44)/ERK(42/44). **D** quantitative analysis of the protein expressions of p-CREB/CREB. Data were expressed as the mean ± SEM. *p < 0.05, compared with the BCCAO/CRS + vehicle group

As shown in Fig. 6, the western blot results indicated that the BCCAO + vehicle group had lower protein expressions of p-TrkB/TrkB, p-ERK(42/44)/ERK(42/44) and p-CREB/CREB than those in the control group (*p* < 0.01 for p-CREB/CREB). The differences in the protein expressions of p-TrkB/TrkB, p-ERK(42/44)/ERK(42/44) were not statistically significant between the control group and the BCCAO + vehicle group. The administration of M-GMDZD at medium dose effectively up-regulated the protein levels of p-TrkB/TrkB (*p* < 0.05), as compared with the BCCAO/CRS + vehicle group. The treatments of M-GMDZD at all doses and PC significantly increased the protein levels of p-ERK(42/44)/ERK(42/44) (*p* < 0.05 for M-GMDZD at low dose; *p* < 0.01 for M-GMDZD at medium and high dose; *p* < 0.001 for PC). Treatment with M-GMDZD at high dose significantly increased the phosphorylation of CREB (*p* < 0.05), when compared with the BCCAO/CRS + vehicle group.

## Discussion

This study demonstrates for the first time that M-GMDZD exhibits potent anti-BPSD properties, including sedative, anxiolytic, and antidepressant-like effects in animal models, underscoring its potential as an innovative, experimentally-tested, knowledge-based Chinese herbal preparation for BPSD management.

**Fig. 7:**
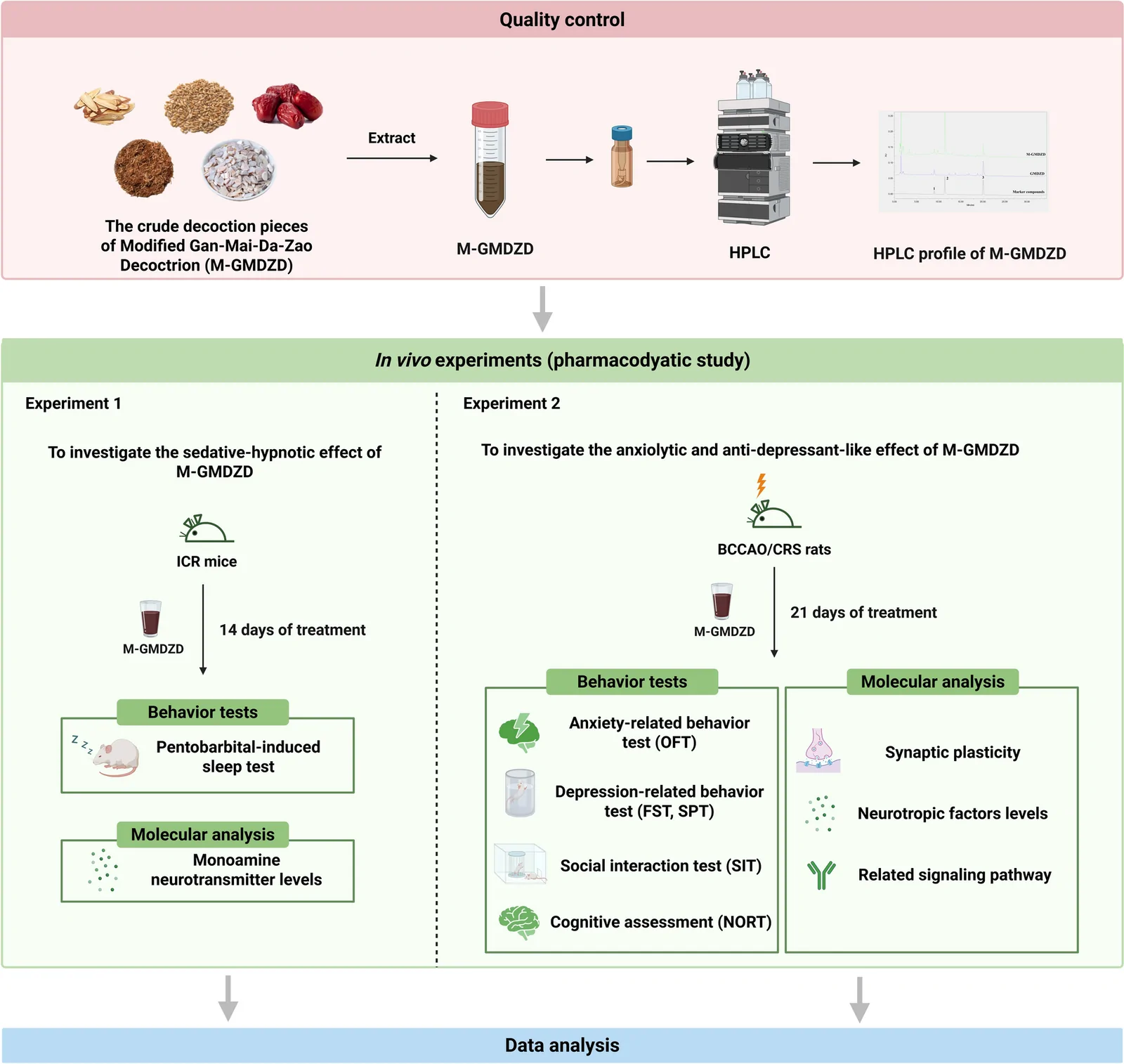
The experimental flowchart

BPSD affects over 90% of dementia patients, but effective therapeutics is currently lacking for this condition. Given that the multifactorial pathogenesis of BPSD, drugs with multi-target ability are perceived as a more promising therapeutic strategy for BPSD, and multi-target therapeutic approach could overcome the poor efficacy seen for the conventional one-target-one-compound drug discovery strategy. Owing to its nature of multiple components, multi-targets and multi-pathways characteristics, CM has garnered increasing attention as a potential therapeutic modality for BPSD [26, 27]. M-GMDZD is an innovative formula modified from GMDZD, a classical herbal formula commonly prescribed by CM practitioners for psychiatric diseases. M-GMDZD incorporates AF, traditionally used for its anxiolytic and mood-regulating properties, and MC, a calcium carbonate-rich marine-derived ingredient with documented sedative and neuroprotective effects, into GMDZD. This modified preparation exemplifies the adaptive nature of CM, where the classical GMDZD is refined through the integration of complementary therapeutic agents while maintaining the foundational principle of “medicine and food sharing the same origin” (Yao-shi-tong-yuan in Chinese). Our two-experiment design systematically evaluates M-GMDZD's therapeutic potential across the BPSD spectrum: Experiment 1 demonstrates sedative-hypnotic effects relevant to sleep-related BPSD symptoms using the pentobarbital-induced sleep model, while Experiment 2 comprehensively evaluates anxiolytic, antidepressant-like, and social interaction effects using the clinically relevant BCCAO/CRS model (Fig. 7). This approach reflects the multifaceted nature of BPSD, where sleep disturbances, depression, anxiety, and social dysfunction often co-occur and require multi-target therapeutic interventions.

Sleep disturbance is one of the most significant symptoms of BPSD [3]. The frequent sleep impairments observed in dementia include persistent short sleep duration, sleep onset delays, excessive nocturnal awakening, and early morning awakening [28, 29]. Notably, sleep abnormalities, including prolonged sleep latency and decreased sleep efficiency, are closely associated with worsening cognitive dysfunction and behavioral symptoms such as depression and anxiety [30, 31]. Thus, effective and timely intervention for sleep disturbances in dementia is essential. In this study, we evaluated whether M-GMDZD treatment could enhance sleep time on top of the hypnotic function of pentobarbital. The pentobarbital-induced sleep model is a well-validated animal model for assessing the drug-induced sleep improvement due to its reliability and reproducibility [32]. Our results demonstrated that while M-GMDZD did not affect sleep latency in the pentobarbital-treated mice, high-dose M-GMDZD administered for 14 days significantly and selectively prolonged the pentobarbital-induced sleep duration, signifying the sedative-hypnotic and sleep-improving properties of M-GMDZD in mice.

To further investigate the mechanisms underlying the sedative-hypnotic effects of M-GMDZD, we evaluated monoamine neurotransmitter levels in the brains of the pentobarbital-treated mice. Monoamine neurotransmitters, including 5-HT, DA, and NE, are closely associated with sleep regulation. Inhibition of 5-HT synthesis can reduce sleep and impair homeostatic responses to sleep deprivation, demonstrating that the serotonergic system promotes sleep [33]. Additionally, 5-HT can also facilitate sleep indirectly by promoting melatonin synthesis [34]. DA and NE play crucial roles in maintaining wakefulness and arousal [35, 36]. The present study showed that M-GMDZD treatment could significantly increase the cerebral 5-HT level and decrease the cerebral DA level in the pentobarbital-treated mice, thereby contributing to sleep enhancement. Our results also reported the lack of significant effects of M-GMDZD on NE and GABA levels in pentobarbital-treated mice, which may be related to our selected experimental model. GABA, as the primary inhibitory neurotransmitter, is indeed crucial for sleep promotion, but pentobarbital itself acts as a GABA_A receptor positive allosteric modulator, potentially masking additional GABAergic effects of M-GMDZD. Similarly, NE's wake-promoting effects through the locus coeruleus may be already suppressed by pentobarbital’s sedative action, limiting our ability to detect M-GMDZD’s modulatory effects on this system. Future studies using natural sleep models or examining neurotransmitter dynamics at different time points could provide more comprehensive insights into M-GMDZD’s effects on the complete neurotransmitter network regulating sleep–wake cycles.

In this study, we used the BCCAO/CRS rat model to evaluate the anxiolytic and antidepressant-like effects of M-GMDZD. The model established in this study first employs a modified two-step ligation procedure for BCCAO, which reduces animal mortality while simulating the progressive cerebrovascular pathology characteristic of neurodegenerative diseases such as vascular dementia and AD. Subsequently, rats are exposed to CRS for 14 days to induce psychological impairments including anxiety and depression. Thus, BCCAO/CRS rat model closely mimics the clinical symptoms seen in BPSD, and accurately imitates the pathophysiological process of BPSD [24]. The present study demonstrated that M-GMDZD treatment could significantly alleviate the anxiety-related behavioral impairments, ameliorate depression-like deficits, attenuate recognition memory impairments and improve social interaction in BCCAO/CRS rats, as assessed by a battery of behavioral tests including SIT, OFT, NORT, FST, and SPT (Fig. 3). These behavioral tests directly correspond to clinically relevant BPSD domains: OFT for anxiety-related behavior, FST for depression-like behavior, SPT for anhedonia (a key feature of depression in dementia), SIT for social dysfunction. Then we further evaluated the effects of M-GMDZD on neurotrophic factors in the brains of BCCAO/CRS rats. Neurotrophic factors, such as BDNF, NGF and NT-3, have been reported to play essential roles in BPSD manifestations, including anxiety, depression and aggression [37]. Our experimental findings showed that M-GMDZD significantly increased the cerebral NT-3 level while having no effect on BDNF and NGF levels in BCCAO/CRS rats, suggesting that anxiolytic and antidepressant-like effects of M-GMDZD may partly attributed to the NT-3-mediated neurotrophic mechanisms. Consistent with these results, previous studies have identified NT-3 as a key regulator of synaptic plasticity in the hippocampus, with critical implications for cognitive functions and BPSD symptoms [38, 39]. Synaptic plasticity dysfunction, particularly involving impairments in NMDA receptor subunits (GluN2A and GluN2B) and AMPA receptor subunits (GluA1 and GluA2), contributes to BPSD symptoms like agitation, anxiety and depression [40, 41]. Conversely, interventions that enhance synaptic plasticity, such as physical exercise, may potentially reduce BPSD severity [42]. Moreover, NT-3 can interact with NMDA receptors to promote synaptic activity and potentiate AMPA receptor-mediated transmission [43]. Thus, we subsequently investigated whether M-GMDZD was able to ameliorate synaptic deficits in BCCAO/CRS rats. Our results demonstrated that M-GMDZD could markedly increase the protein expressions of GluN2A, GluN2B, and GluA2 in the brains of BCCAO/CRS rats (Fig. 6A), indicating that anti-BPSD effect of M-GMDZD is, at least partially, related to its synaptic plasticity improving property. We also found that the TrkB/ERK/CREB pathway is critically associated with synaptic dysfunction in BPSD, serving as a convergence point for neurotrophin (including NT-3) signaling deficits that underlie non‐cognitive symptoms. It was reported that reduced NT-3 and BDNF levels in BPSD models diminish Trk receptor engagement, leading to impaired activation of the Ras/MAPK (ERK) cascade [37]. Insufficient ERK phosphorylation attenuates the CREB-mediated transcription of synaptic plasticity genes, resulting in behavioral symptoms [44, 45]. Our present results showed that M-GMDZD significantly up-regulated the protein expressions of p-TrkB/TrkB, p-ERK(42/44)/ERK(42/44) and p-CREB/CREB in BCCAO/CRS rats, suggesting that M-GMDZD exerts anti-BPSD effects through activation of TrkB/ERK/CREB signaling pathway. Collectively, these findings indicate that M-GMDZD treatment could alleviate BPSD symptoms in BCCAO/CRS rats by restoring synaptic plasticity via the TrkB/ERK/CREB pathway.

However, our current study may have several limitations. First, we did not conduct pharmacokinetic studies to determine which components reach systemic circulation and their bioactive concentrations. Second, we did not perform individual component testing to establish which herbs drive the observed effects. Additionally, the western blot analyses in this study were based on three biological replicates, which is appropriate for exploratory mechanistic profiling but may limit the power to definitively conclude the absence of changes in proteins that did not reach statistical significance. These non-significant findings should therefore be interpreted with caution, and future studies incorporating larger sample sizes will be necessary to further validate and expand current findings.

**Fig. 8:**
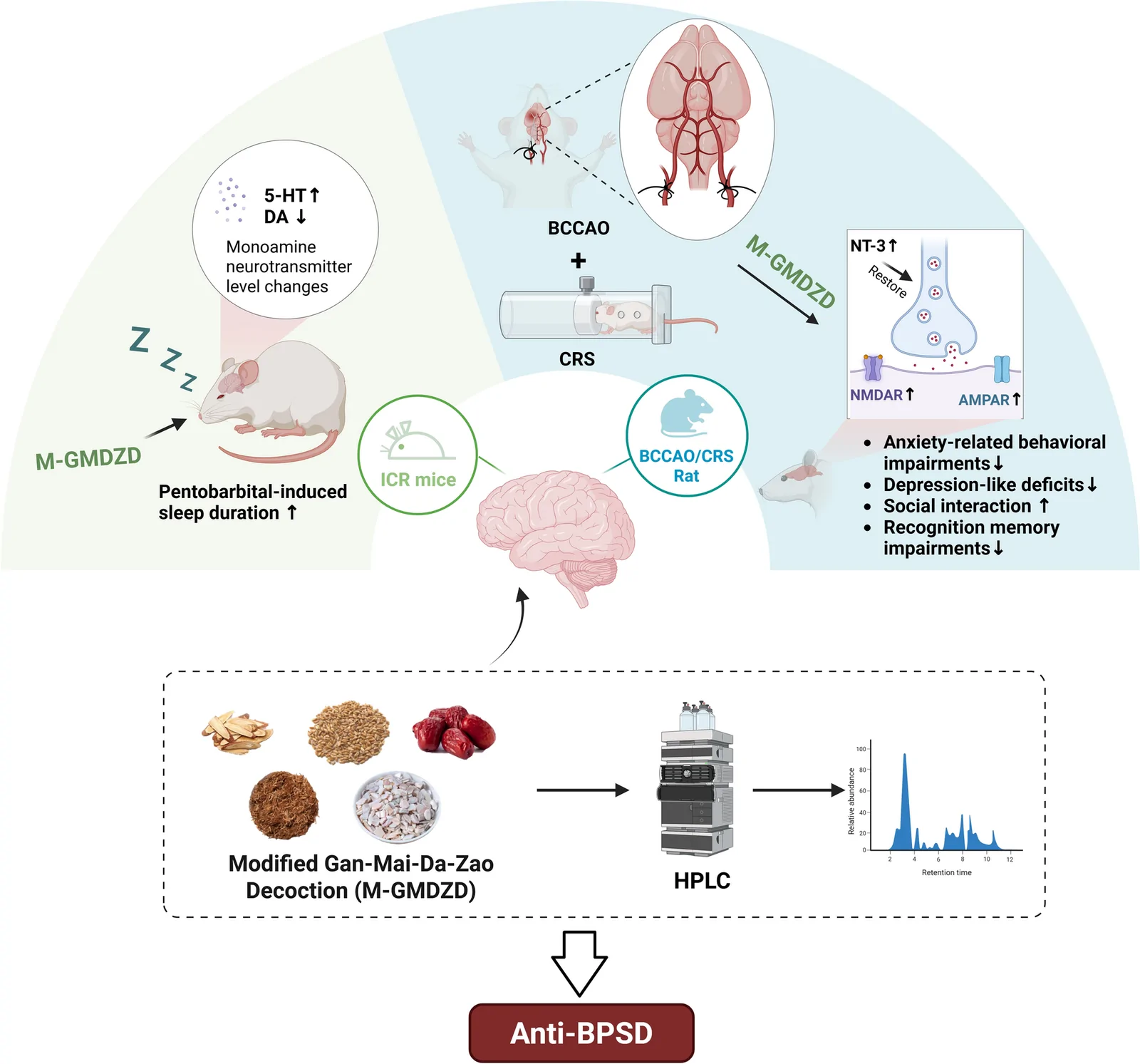
The schematic diagram summarizing the proposed mechanisms underlying the anti-BPSD effects of M-GMDZD

To conclude, this study reports for the first time that M-GMDZD possesses robust anti-BPSD efficacy as evidenced by its sedative, anxiolytic, and antidepressant-like activities in experimental models, signifying its potential as a novel therapeutic approach for BPSD (Fig. 8).

## Supplementary Information

Supplementary material 1.

Supplementary material 2.

Supplementary material 3.

## Data Availability

All data supporting the conclusions of this article are included with this article.

## Abbreviations

p5-HT: 5-hydroxytryptamine
AD: Alzheimer’s disease
AF: Albiziae Flos
BCCAO/CRS: Bilateral common carotid arteries occlusion combined with chronic restraint stress
BPSD: Behavioral and psychological symptoms of dementia
CCA: Common carotid artery
CM: Chinese medicine
DA: Dopamine
DEG: Differentially expressed genes
FST: Forced swim test
GABA: γ-aminobutyric acid
GMDZD: Gan-Mai-Da-Zao Decoction
GRR: Glycyrrhizae Radix et Rhizoma
HF: Hordei Fructus
HPLC: High-performance liquid chromatography
JF: Jujubae Fructus
MC: Margaritifera Concha
M-GMDZD: Modified Gan-Mai-Da-Zao Decoction
NE: Norepinephrine
NGF: Nerve growth factor
NORT: Novel recognition test
NT-3: Neurotrophin-3
OFT: Open field test
PVDF: Polyvinyl difluoride
RCTs: Randomized clinical trials
SIT: Social interaction test
SPT: Sucrose preference test
