# Supplementary material for: Modified Gan-Mai-Da-Zao decoction for behavioral and psychological symptoms of dementia: preclinical efficacy and molecular mechanisms
Source: Chin Med. 2026 Aug 20;21:210. doi: 10.1186/s13020-026-01415-y (PMC13491767; doi:10.1186/s13020-026-01415-y)
Supplement: Supplementary file 1 [file 13020_2026_1415_MOESM3_ESM.pdf]

GluN2A

Rep1 **Rep2** **Rep3** **Rep4** **Rep5**

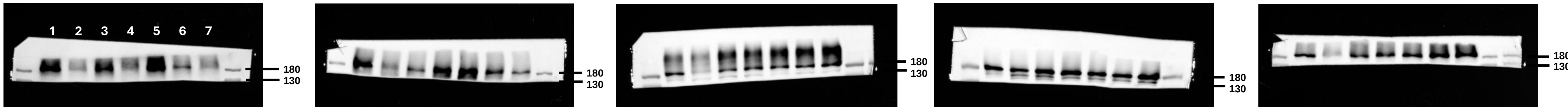

GluA1

Rep1 **Rep2** **Rep3** **Rep4** **Rep5**

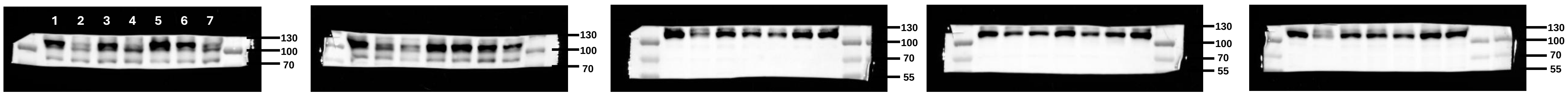

GAPDH (for GluN2A and GluA1)

Rep1 **Rep2** **Rep3** **Rep4** **Rep5**

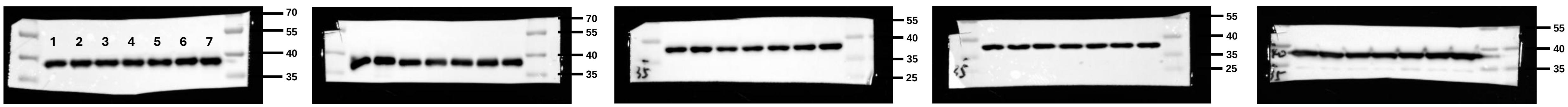

The whole membrane

Rep1 **Rep2** **Rep3** **Rep4** **Rep5**

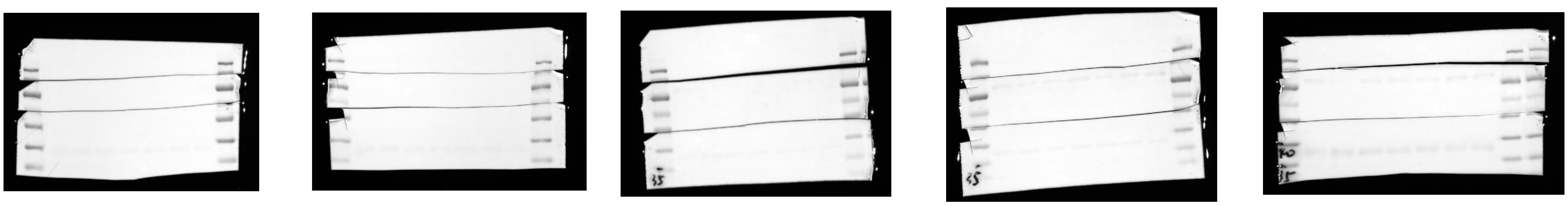

***Each whole membrane was cut into three sections and incubated separately with the corresponding antibodies, including GluN2A, GluA1 and GAPDH.**

Note: 1, Control; 2, BCCAO/CRS; 3, BCCAO/CRS + GMDZD; 4, BCCAO/CRS + M-GMDZD-L; 5, BCCAO/CRS + M-GMDZD-M; 6, BCCAO/CRS + M-GMDZD-H; 7, BCCAO/CRS + PC.

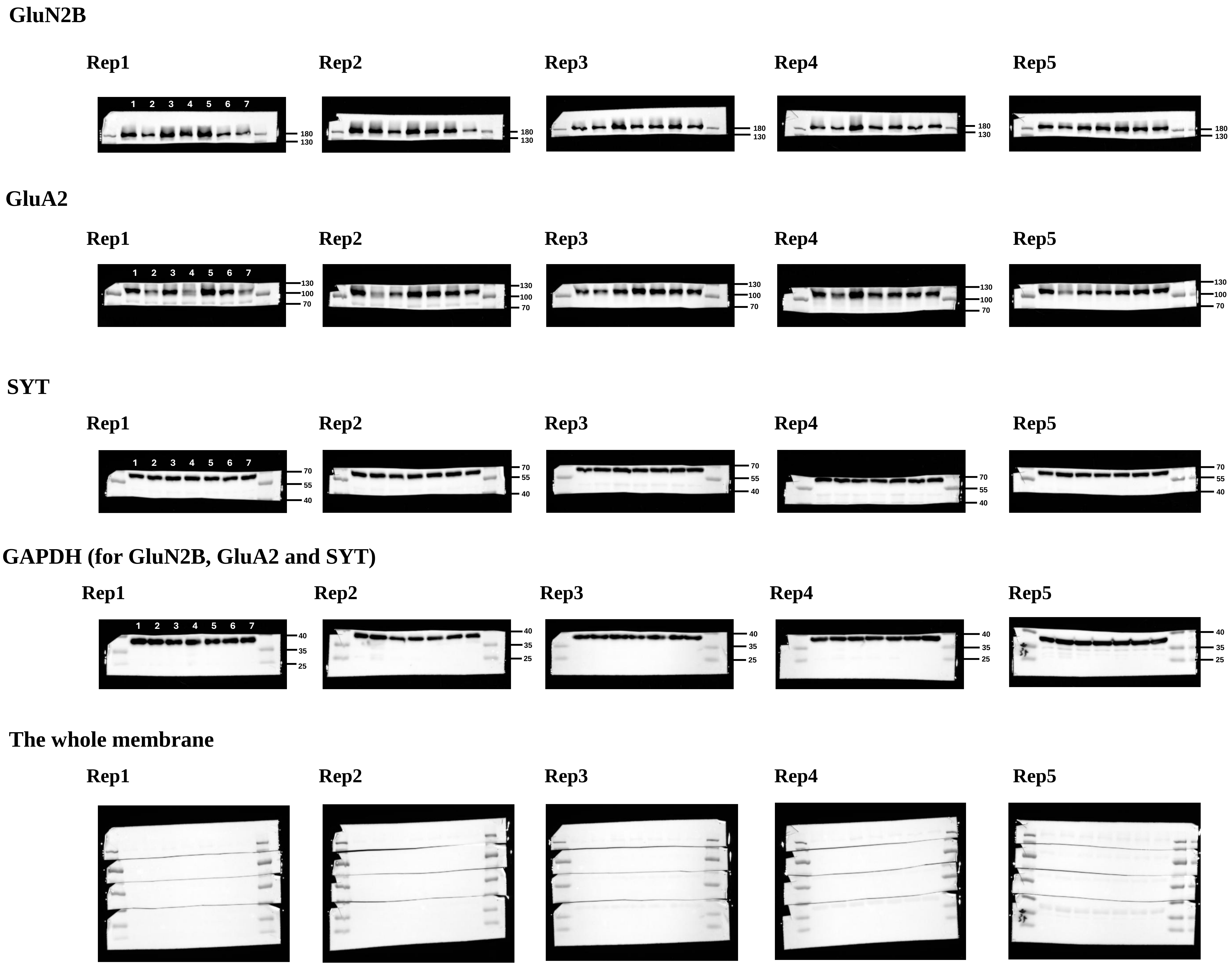

***Each whole membrane was cut into four sections and incubated separately with the corresponding antibodies, including GluN2B, GluA2, SYT and GAPDH.**

Note: 1, Control; 2, BCCAO/CRS; 3, BCCAO/CRS + GMDZD; 4, BCCAO/CRS + M-GMDZD-L; 5, BCCAO/CRS + M-GMDZD-M; 6, BCCAO/CRS + M-GMDZD-H; 7, BCCAO/CRS + PC.

SYN1

Rep1 Rep2 Rep3 Rep4 Rep5

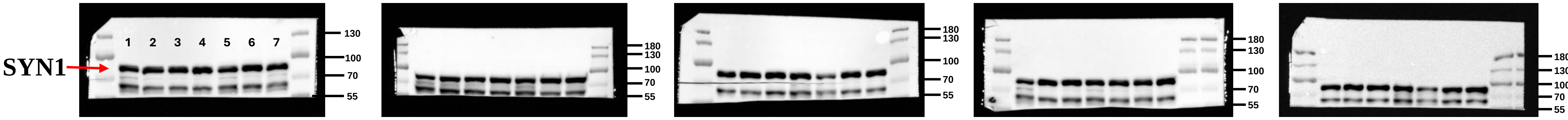

GAPDH

Rep1 Rep2 Rep3 Rep4 Rep5

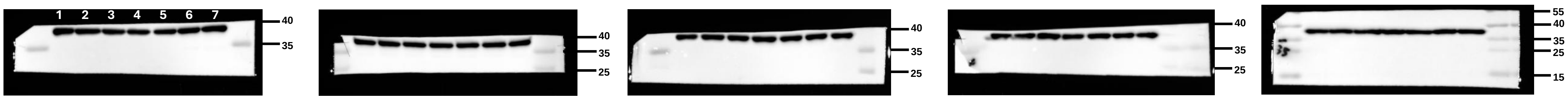

The whole membrane

Rep1 Rep2 Rep3 Rep4 Rep5

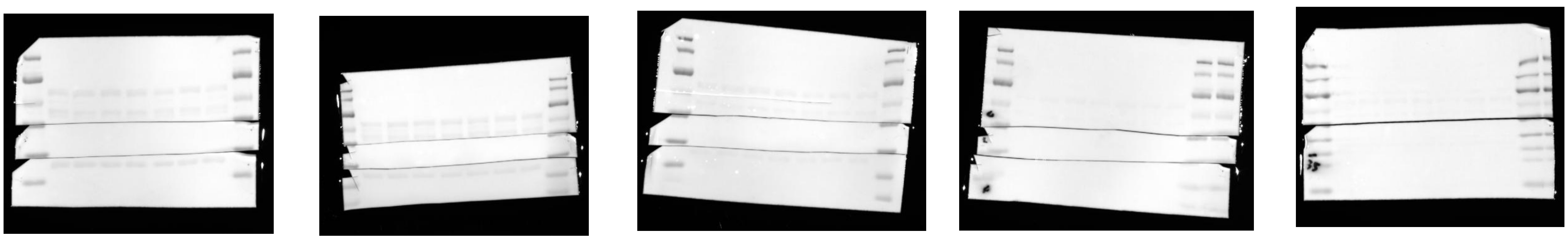

***Each whole membrane was cut and incubated separately with the corresponding antibodies, including SYN1 and GAPDH.**

Note: 1, Control; 2, BCCAO/CRS; 3, BCCAO/CRS + GMDZD; 4, BCCAO/CRS + M-GMDZD-L; 5, BCCAO/CRS + M-GMDZD-M; 6, BCCAO/CRS + M-GMDZD-H; 7, BCCAO/CRS + PC.

PSD93

Rep1 Rep2 Rep3 Rep4 Rep5

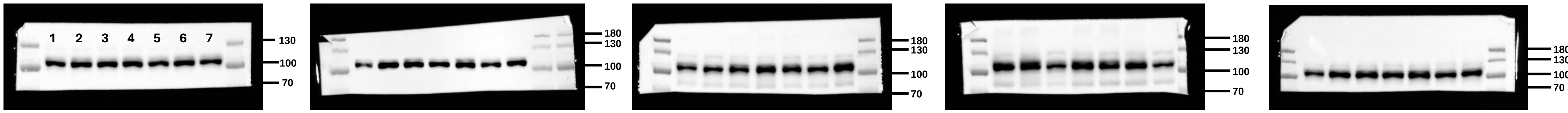

GAPDH

Rep1 Rep2 Rep3 Rep4 Rep5

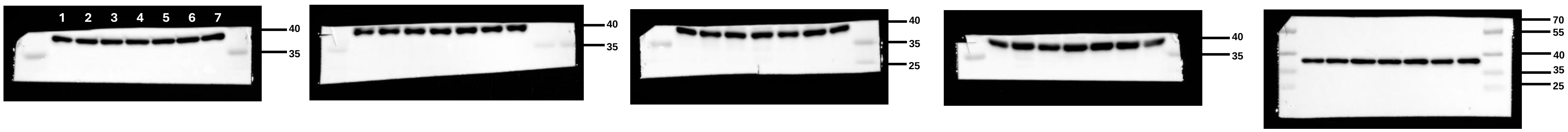

The whole membrane

Rep1 Rep2 Rep3 Rep4 Rep5

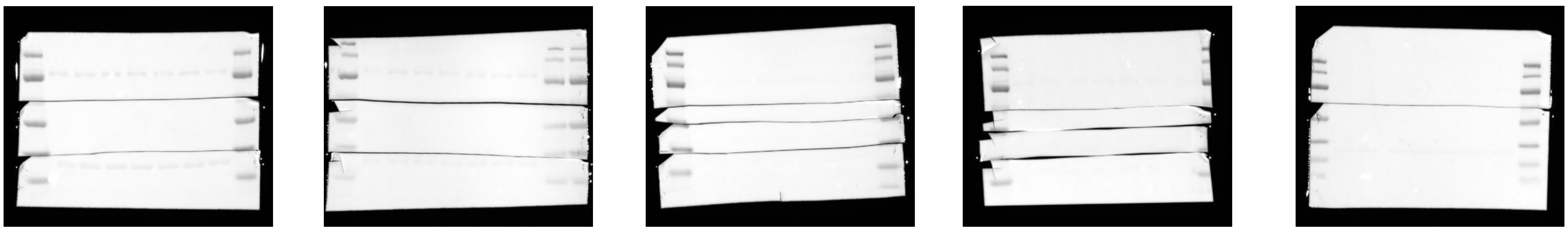

***Each whole membrane was cut and incubated separately with the corresponding antibodies, including PSD93 and GAPDH.**

Note: 1, Control; 2, BCCAO/CRS; 3, BCCAO/CRS + GMDZD; 4, BCCAO/CRS + M-GMDZD-L; 5, BCCAO/CRS + M-GMDZD-M; 6, BCCAO/CRS + M-GMDZD-H; 7, BCCAO/CRS + PC.

PSD95

Rep1 Rep2 Rep3 Rep4 Rep5

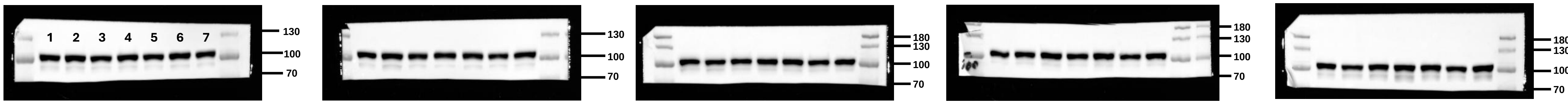

GAPDH

Rep1 Rep2 Rep3 Rep4 Rep5

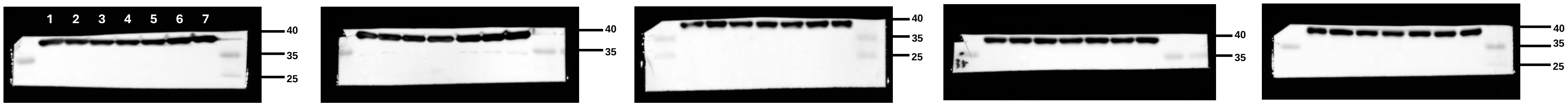

The whole membrane

Rep1 Rep2 Rep3 Rep4 Rep5

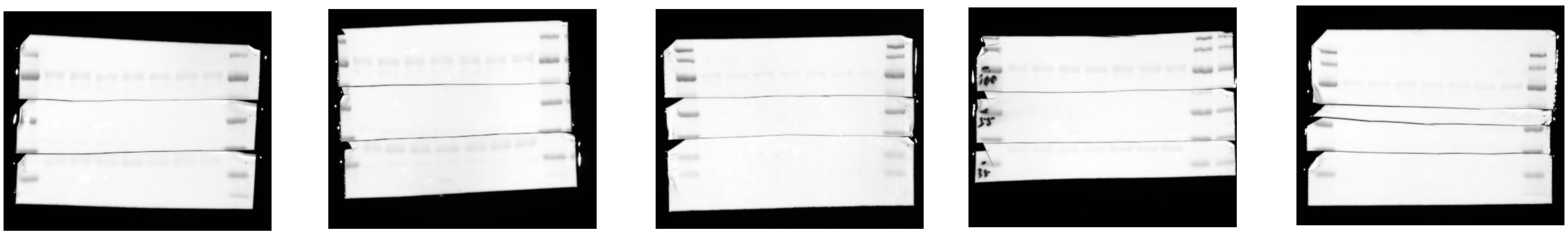

***Each whole membrane was cut and incubated separately with the corresponding antibodies, including PSD95 and GAPDH.**

Note: 1, Control; 2, BCCAO/CRS; 3, BCCAO/CRS + GMDZD; 4, BCCAO/CRS + M-GMDZD-L; 5, BCCAO/CRS + M-GMDZD-M; 6, BCCAO/CRS + M-GMDZD-H; 7, BCCAO/CRS + PC.

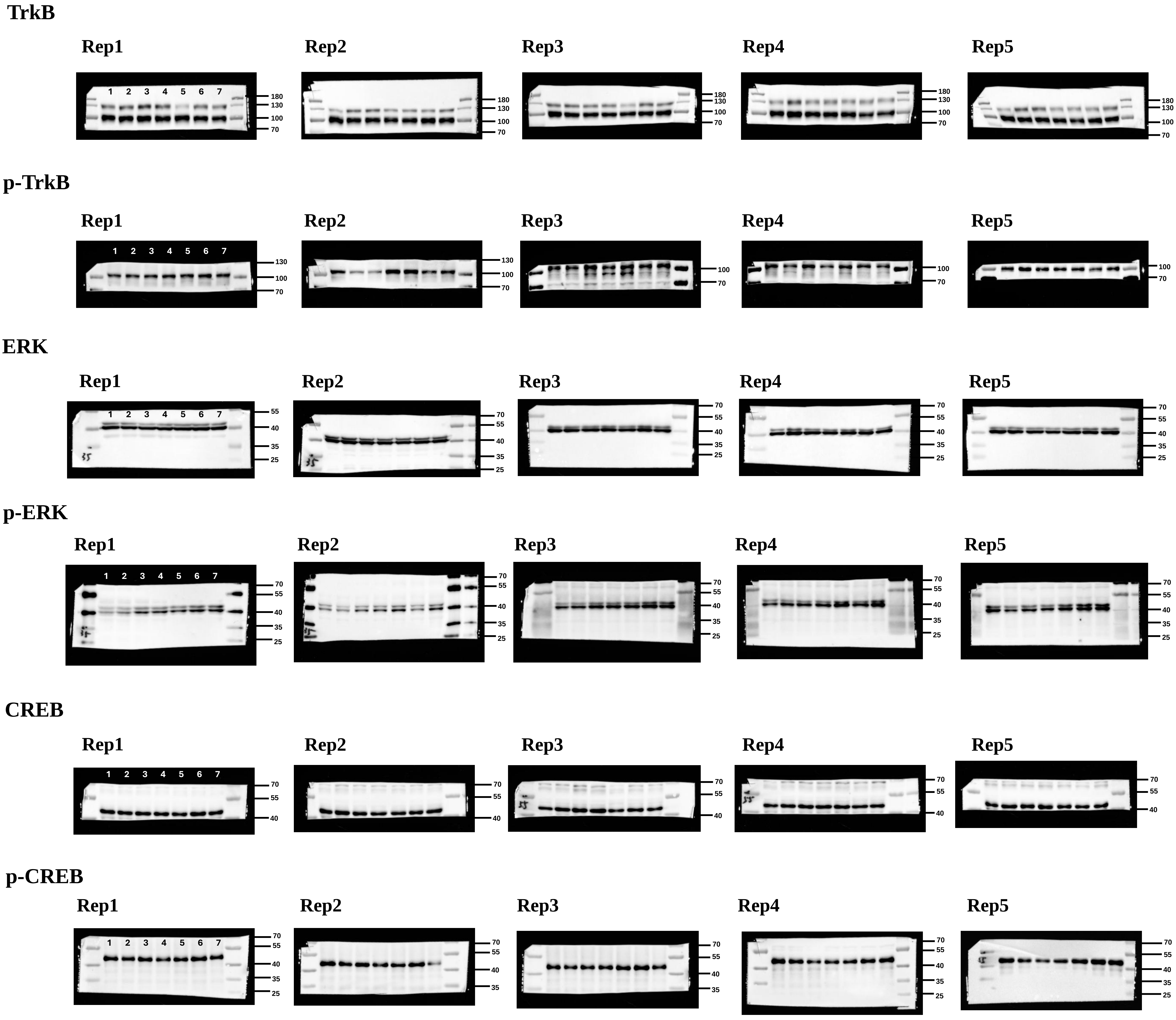

Note: 1, Control; 2, BCCAO/CRS; 3, BCCAO/CRS + GMDZD; 4, BCCAO/CRS + M-GMDZD-L; 5, BCCAO/CRS + M-GMDZD-M; 6, BCCAO/CRS + M-GMDZD-H; 7, BCCAO/CRS + PC.
