# Supplementary material for: Modified Gan-Mai-Da-Zao decoction for behavioral and psychological symptoms of dementia: preclinical efficacy and molecular mechanisms
Source: Chin Med. 2026 Aug 20;21:210. doi: 10.1186/s13020-026-01415-y (PMC13491767; doi:10.1186/s13020-026-01415-y)
Supplement: Supplementary file 2 [file 13020_2026_1415_MOESM2_ESM.docx]

**Fig. S1.** RNA-seq analysis reveals neuronal signaling- and synaptic function-related transcriptomic alterations in the M-GMDZD group. A, Volcano plot of differentially expressed genes (DEGs), with representative genes related to neuroactive signaling and synaptic regulation highlighted. B, Gene Ontology (GO) enrichment analysis of DEGs. C, KEGG pathway classification of DEGs. D, KEGG pathway enrichment analysis of DEGs.
